# Why Target Innate Immune Cells in Cancers?

**DOI:** 10.3390/cancers13040690

**Published:** 2021-02-09

**Authors:** Mary Poupot

**Affiliations:** 1 Centre de Recherches en Cancérologie de Toulouse, Inserm UMR1037, 31037 Toulouse, France; mary.poupot@inserm.fr; 2 Université Toulouse III Paul-Sabatier, 31400 Toulouse, France 3. ERL 5294 CNRS, 31037 Toulouse, France

The immune system is a smart way to fight cancer, with its precise targeting of cancer cells sparing healthy cells. It can also adapt continuously and dynamically according to the tumor evolution and develop a memory against cancer cells. However, when the immune system can no longer fight cancer cells, the tumor wins and develops. For twenty years, the cancer immunoediting theory proposed by Schreiber and Smyth has played an important part in cancer therapies [1]. Indeed, the immune system plays a dual role suppressing or promoting tumor growth. This mechanism of extrinsic tumor suppressor is based on three sequential phases: elimination, equilibrium and escape. The elimination phase consists of the destruction of cancer cells by innate and adaptive immunity. If a rare cancer cell variant is not destroyed, it could enter an equilibrium in which its outgrowth is prevented by adaptive immunologic mechanisms, maintaining a state of functional dormancy. However, some tumor cell variants could emerge which are able to escape the adaptive immune system and induce an immunosuppressive state within the tumor microenvironment (TME). In this phase, all cells in the TME, including cancer cells, play a role in the tumor development. Some infiltrated immune cells contribute, therefore, to maintaining a favorable environment for cancer cells, promoting their survival, immune escape, invasive capabilities, and therapeutic resistance, as well as angiogenesis. Within the tumor, immune cells can be represented by adaptive and innate immune cells. Among these innate immune cells, we can find innate lymphoid cells (ILCs), eosinophils, basophils, mast cells and phagocytic cells, such as macrophages, neutrophils, and dendritic cells (DC).

The regular job of all these innate immune cells is to protect the body against pathogens such as bacteria, viruses, parasites, or fungi, and to eliminate modified cells, apoptotic cells, debris, and cancer cells. They represent the first line of defense, which then helps with adaptive immunity. In cancer, we do not know if innate immune cells are the first to reach the tumor site before the adaptive immune cells. However, it is well known that there are, in each organ/tissue type, resident immune cells such as macrophages [2], which can therefore be on site at the tumor initiation. Resident immune cells, as well as those recruited, are able to be involved in the elimination phase but also in the escape phase, during which they can be turned in the favor of the tumor development. This pro-tumor role is facilitated by the plasticity of these innate immune cells then exhibiting varied phenotypes and functions, depending on the type of the innate immune cell considered and the type of tumors. All innate immune cells inside the TME can be involved in processes favoring tumor cell survival, proliferation, motility and invasive potential, resistance to treatment and to immune attacks, epithelial–mesenchymal transition, and angiogenesis. However, the mechanisms of these processes can differ according to the immune cell considered, to the tissue, and to the stage of the tumor [3,4,5]. These diverse mechanisms can involve cell–cell interactions via different membrane molecules at the surface of immune cells and cancer cells. These interactions can be established between immune cells and cancer cells or between immune cells themselves. Soluble factors such as chemokines, prostaglandins, enzymes, growth factors, interleukins and other cytokines, and molecules such as reactive oxygen species or adenosine, for instance, are also involved in the pro-tumor functions of innate immune cells in the TME. These soluble factors are produced by immune cells to have a direct effect on cancer cells, or an indirect effect targeting other immune cells to abrogate immune attack or to enhance immunosuppressive functions.

Target innate immune cells inside the TME can therefore be a good way to limit tumor progression. This targeting can be drastic by the depletion of these cells using antibodies or chemical drugs, which are more or less specific. However, the plasticity of these cells allows the reversion of their pro-tumor functions in anti-tumor functions which can be, of course, reversible, and with a relative specificity.

Targeting these cells is therefore a real challenge today facing classical therapies that are, in some cases, insufficient or inappropriate. The combination of new targeting immunotherapies with classical chemo- or immunotherapies could be a solution. However, even though many studies propose antibodies or chemical molecules to target innate immune cells, new innovative strategies have to be developed in order to be more specific and efficient. The Special Issue “Targeting the innate immune cells in cancer” aims to highlight studies developing novel strategies to target innate immune cells in order to promote the inhibition of their pro-tumor functions.
